# Restoring Inflammatory Mediator Balance after Sofosbuvir-Induced Viral Clearance in Patients with Chronic Hepatitis C

**DOI:** 10.1155/2018/8578051

**Published:** 2018-05-27

**Authors:** Geórgia Nascimento Saraiva, Natalia Fonseca do Rosário, Thalia Medeiros, Paulo Emílio Côrrea Leite, Gilmar de Souza Lacerda, Thaís Guaraná de Andrade, Elzinandes Leal de Azeredo, Petronela Ancuta, Jorge Reis Almeida, Analúcia Rampazzo Xavier, Andrea Alice Silva

**Affiliations:** ^1^Laboratório Multiusuário de Apoio à Pesquisa em Nefrologia e Ciências Médicas, Faculdade de Medicina, Hospital Universitário Antônio Pedro, Universidade Federal Fluminense, Niterói, Brazil; ^2^Laboratório de Ultraestrutura Celular Hertha Meyer, Instituto de Biofísica Carlos Chagas Filho, Universidade Federal do Rio de Janeiro, Rio de Janeiro, Brazil; ^3^Departamento de Patologia, Faculdade de Medicina, Universidade Federal Fluminense, Niterói, Brazil; ^4^Centro de Referência de Tratamento em Hepatites/HUAP, Serviço de Gastroenterologia, Departamento de Medicina Clínica, Faculdade de Medicina, Universidade Federal Fluminense, Niterói, Brazil; ^5^Laboratório de Imunologia Viral, Instituto Oswaldo Cruz, Fundação Oswaldo Cruz, Rio de Janeiro, RJ, Brazil; ^6^Department of Microbiology, Infectiology and Immunology, Centre de Recherche du CHUM, Université de Montréal, Montreal, Canada

## Abstract

This study aimed at analyzing circulating levels of inflammatory and profibrogenic cytokines in patients with hepatitis C virus (HCV) chronic infection undergoing therapy with direct-acting antiviral agents (DAA) and correlating these immune biomarkers with liver disease status. We studied 88 Brazilian monoinfected chronic hepatitis C patients receiving interferon- (IFN-) free sofosbuvir-based regimens for 12 or 24 weeks, followed-up before therapy initiation and three months after the end of treatment. Liver disease was determined by transient elastography, in addition to APRI and FIB-4 indexes. Analysis of 30 immune mediators was carried out by multiplex or enzymatic immunoassays. Sustained virological response rate was 98.9%. Serum levels of cytokines were increased in HCV-infected patients when compared to control group. CCL-2, CCL-3, CCL-4, CXCL-8, CXCL-10, IL-1*β*, IL-15, IFN-*γ*, IL-4, IL-10, TGF-*β*, FGFb, and PAI-1 decreased significantly after antiviral therapy, reaching values similar to noninfected controls. TGF-*β* and suPAR levels were associated with fibrosis/cirrhosis. Also, we observed amelioration in hepatic parameters after DAA treatment. Together, our results suggest that viral control induced by IFN-free DAA therapy restores inflammatory mediators in association with improvement in liver function.

## 1. Introduction

Chronic hepatitis C is an inflammatory liver disease caused by hepatitis C virus (HCV) persistent infection. The global prevalence of HCV infection was estimated in 1.0% of population or 71.1 million of individuals worldwide [1]. Most patients develop chronic hepatitis C, characterized by presence of liver fibrosis, which can lead to cirrhosis, hepatocellular carcinoma, and even death [2]. Currently, second-generation direct-acting antiviral agents (DAA) have been used for chronic hepatitis C treatment. The association of sofosbuvir (SOF) with daclatasvir (DCV) or simeprevir (SMV), with or without ribavirin (RBV), directly inhibits viral replication acting on NS5B, NS3/4A, and/or NS5A enzymes [3]. SOF-based antiviral therapy guarantees efficacy in HCV eradication in approximately 90% of cases and is associated with mild to moderate adverse effects [1, 2, 4].

Viral replication takes place mainly in hepatocytes and its persistence induces an intense inflammatory response that might result in liver fibrosis, which is a process orchestrated by numerous immune mediators and by induction of interferon-stimulated genes [5–7]. Also, immune and hepatic cells contribute to viral control, establishment and maintenance of inflammation, and progression to liver fibrosis or tissue regeneration [8]. Overall, studies describe an increase in serum cytokine levels in chronic hepatitis C patients, when compared with healthy individuals [9–12]. Therefore, exploiting circulating cytokine array and noninvasive fibrotic biomarkers after interferon- (IFN-) free antiviral therapy with second-generation DAA may contribute to a better understanding about the pattern of these mediators in chronic hepatitis C pathogenesis, progression of liver disease, therapeutic responses, and even persistent infection pathways.

Previous studies suggest that both liver stiffness and inflammatory parameters could improve after HCV eradication [13–15]. On the other hand, the occurrence of downregulation of inflammatory and fibrotic biomarkers in association with HCV eradication after antiviral therapy is still unclear. Some authors demonstrated an increase in inflammatory mediators' serum levels after the end of IFN-based treatment [16–20]. Other studies reported decreased inflammatory biomarkers in plasma of chronic hepatitis C patients after IFN-free regimens including the newer DAA, although normalization was not reached [21, 22]. Recently, a study showed that chronic HCV infection disrupts the milieu of soluble inflammatory mediators even after viral clearance with DAA treatment, suggesting that the inflammatory changes are not fully reversible upon clearance of viral infection [11]. These conflicting results in literature could be due to host (age and immune response) and/or viral factors (genotype and viral load) or even according to treatment regimen (IFN-free or not) [12, 23, 24].

Hence, the present study aimed at analyzing circulating levels of inflammatory and profibrogenic cytokines in chronic hepatitis C patients undergoing DAA therapy. In addition, we evaluated correlations between immune biomarkers and liver disease stage.

## 2. Material and Methods

### 2.1. Study Design

We conducted an observational and prospective study with Brazilian monoinfected chronic hepatitis C patients receiving IFN-free SOF-based regimens. From December 2015 to April 2017, HCV-infected patients with indication for DAA therapy were recruited at the Hepatitis Treatment Center of Antonio Pedro University Hospital (HUAP), located in Niteroi city (RJ, Brazil). The study was approved by the Research Ethics Committee of the Federal Fluminense University (number 35033514.5.0000.5243), which was conducted in accordance to the principles expressed in the Declaration of Helsinki. Written informed consent was obtained from all enrolled subjects.

Chronic hepatitis C patients were followed before DAA therapy initiation and three months after the end of therapy, when individuals could reach sustained virological response (SVR). The treatment consisted of daily doses of SOF (400 mg) in association with SMV (150 mg) or DCV (60 mg), for 12 or 24 weeks. RBV (13–15 mg/kg) was added to therapeutic regimens when patients presented predictors of poor response to treatment [2]. No patient included in this study received IFN during DAA therapy. Diagnosis, prescription, and clinical monitoring were performed by clinical group from Hepatitis Treatment Center (Niteroi, Brazil).

Thirteen HCV noninfected volunteers were included in the study as a control group.

### 2.2. Data Collection and Blood Sampling

Clinical and laboratory follow-ups were performed before treatment initiation (baseline data) and at 12 weeks after the end of treatment (SVR). Clinical data—such as HCV genotype, viral load, and current or prior treatments—were obtained from patient's charts and confirmed with attending clinicians.

Venous blood collection was performed for the inflammatory and fibrotic biomarker analysis. Blood samples were collected in the morning after 12 h overnight fasting in Vacutainer® blood collection tubes with serum-clotting activator (Becton Dickson, USA) and tubes containing 3.2% of sodium citrate (Becton Dickson, USA) to obtain serum and plasma, respectively. Blood samples were centrifugated (1210 ×g/15 min) at room temperature using LS-3 Centrifuge® (Celm, São Paulo, Brazil). Samples were stored at −80°C for posterior analysis.

### 2.3. Liver Disease Staging

Fibrosis or cirrhosis status was determined by hepatic transient elastography (FibroScan®, Echosens, France), a noninvasive technique that measures liver stiffness (F2: moderated fibrosis ≥ 7.1 kPa; F3: advanced fibrosis ≥ 9.5 kPa; F4: cirrhosis ≥ 12.5 kPa) [25]. The procedure was performed by a single well-trained and experienced gastroenterologist.

Alanine aminotransferase (ALT), aspartate aminotransferase (AST), gamma-glutamyl transferase (GGT) and albumin were routinely measured in RXL Max Clinical Chemistry System® (Siemens, Newark, Delaware, USA). Platelet count and prothrombin time (PT) were assessed using the Coulter LH750® (Beckman Coulter, California, USA) and Sysmex® CA-1500 System (Sysmex America Inc., Lincolnshire, Illinois, USA) equipments, respectively. International normalized ratio (INR) was calculated. All tests were performed in the Department of Pathology, HUAP, UFF (Brazil).

Aiming to complement the analysis of liver disease status, platelet ratio index (APRI) and fibrosis- (FIB-) 4 indexes were calculated using simple mathematical formulas previously described [26, 27], for APRI: (AST [level]/AST [upper limit of normal]/platelet count [10^9^/L]) × 100 and for FIB-4: age [years] × (AST [IU/L]/platelet count [expressed as platelets × 10^9^/L] × ALT^1/2^ [IU/L]).

### 2.4. Multiplex Immunoassay

Multiplex bead assay was carried out by Bio-Plex Magpix® commercial kit and reading apparatus (Biorad Laboratories Inc., Hercules, California, USA) according to the manufacturer's recommendation and as previously described [28]. The “xMAP” magnetic technology is based on microspheres that enable the detection of 27 different circulating proteins. Serum levels of the following cytokines were analyzed: chemokine monocyte chemoattractant protein 1 (MCP-1/CCL-2); macrophage inflammatory protein 1*α* (MIP-1α/CCL-3); macrophage inflammatory protein 1*β* (MIP-1*β*/CCL-4); chemokine C-C ligand 5 (RANTES/CCL-5); chemokine C-C ligand 11 (eotaxin-1/CCL-11); interferon gamma-induced protein 10 (IP-10/CXCL-10); granulocyte-macrophage colony-stimulation factor (GM-CSF/CSF-2); granulocyte colony-stimulating factor (G-CSF/CSF-3); basic fibroblast growth factor (FGFb); interferon *γ* (IFN-*γ*); interleukin 1 receptor antagonist (IL-1ra); interleukin 1*β* (IL-1*β*); IL-2, -4, -5, -6, -7, -8 (CXCL-8), -9, -10, -12p70, -13, −15, and -17; platelet-derived growth factor BB (PDGF-BB), tumor necrosis factor (TNF), and vascular endothelial growth factor (VEGF). All samples were tested in duplicate.

### 2.5. Enzyme-Linked Immunosorbent Assays

Commercially available ELISA kits were performed in 96-well clear plates to quantify serum levels of transforming growth factor *β* (TGF-*β*, Invitrogen, Thermo Fisher Scientific, USA), plasminogen activator inhibitor type 1 (PAI-1, PeproTech, USA), FGFb (PeproTech, USA), and soluble urokinase-type plasminogen activator receptor (suPAR, RayBiotech, USA) according to manufacturer's instructions. The reported minimum detectable doses of TGF-*β*, PAI-1, FGFb, and suPAR were 8 pg/mL, 23 pg/mL, 63 pg/mL, and 15 pg/mL, respectively. Absorbance values were obtained after plate reading in spectrophotometer (Spectramax M3 Multi-Mode Microplate Reader®, Molecular Devices, USA) and analyzed using SoftMax Pro 6.0® (Molecular Devices, USA). All samples were tested in duplicate.

### 2.6. Statistical Analysis

Results are expressed as mean ± standard deviation or median (interquartile range). Differences between the control group and chronic hepatitis C patients and comparisons between biomarker levels before and after treatment were analyzed using Mann–Whitney and Wilcoxon tests, respectively. ANOVA or Kruskal-Wallis tests were used for the comparison of circulating biomarkers according to liver disease status, and correlations between parameters were evaluated by linear regression with analysis of Spearman's or Pearson's coefficients. A network of multiple protein-protein interactions was generated using STRING database (http://string-db.org). *P* values less than 0.05 were considered statistically significant. Statistical analysis was carried out using the software Prism 5.0 (GraphPad Prism, San Diego, California, USA) and SPSS Statistics 18.0 (SPSS Inc., Chicago, Illinois, USA).

## 3. Results

### 3.1. Study Population

The demographic and clinical characteristics of 88 chronic hepatitis C patients included in this study are shown in Table 1. Patients presented a mean age of 59.4 (23–73) years, and 69.3% were female. HCV genotype 1 was the most frequently observed (80.7%), and the majority of patients (76.1%) received SOF plus DAC (with or without RBV) therapeutic schemes. In 85.2% of the cases, patients were treated for 12 weeks. The control group was composed of 13 HCV noninfected volunteers who presented a mean age of 43.1 (25–68) years, and 69.2% were females.

In our treatment center, patients with advanced liver injury and treatment-experienced patients had priority for receiving DAA therapy. Thus, 56.8% had already been previously treated with anti-HCV therapies. Also, most patients (80.7%) had cirrhosis, in which 65.9% were categorized in Child-Pugh A classification (compensated cirrhosis) and 14.8% in Child-Pugh B or C (decompensated cirrhosis).

Chronic hepatitis C patients presented serum hepatic parameters overhead reference values at baseline, such as ALT and AST (Table 1). Overall, SVR rate was 98.9% (87/88). The following analyses were performed only with patients who reached the SVR.

### 3.2. Inflammatory and Fibrotic Biomarkers Are Generally Upregulated in Chronic Hepatitis C

In multiplex assay, the levels of eight biomarkers (including CSF-2, IL-5, IL-9, IL-12p70, IL-13, IL-17, FGFb, and TNF) were below the limits of detection in all patients in both baseline and SVR times.  Table 2 shows the comparison of evaluated cytokines between the control group and hepatitis C patients at baseline. Overall, the patients presented increased serum levels of cytokines, chemokines, and growth factors when compared with the control group; however, only CCL-4 (*P* = 0.001), CXCL-10 (*P* = 0.0001), IFN-*γ* (*P* = 0.002), PAI-1 (*P* = 0.008), and suPAR (*P* = 0.02) were statistically different between the groups. In contrast, we observed significantly lower TGF-*β* levels in chronic hepatitis C group (*P* = 0.004) (Table 2).

### 3.3. Inflammatory and Profibrogenic Biomarkers Are Modulated in Chronic Hepatitis C after DAA Therapy

**Figure 1:**
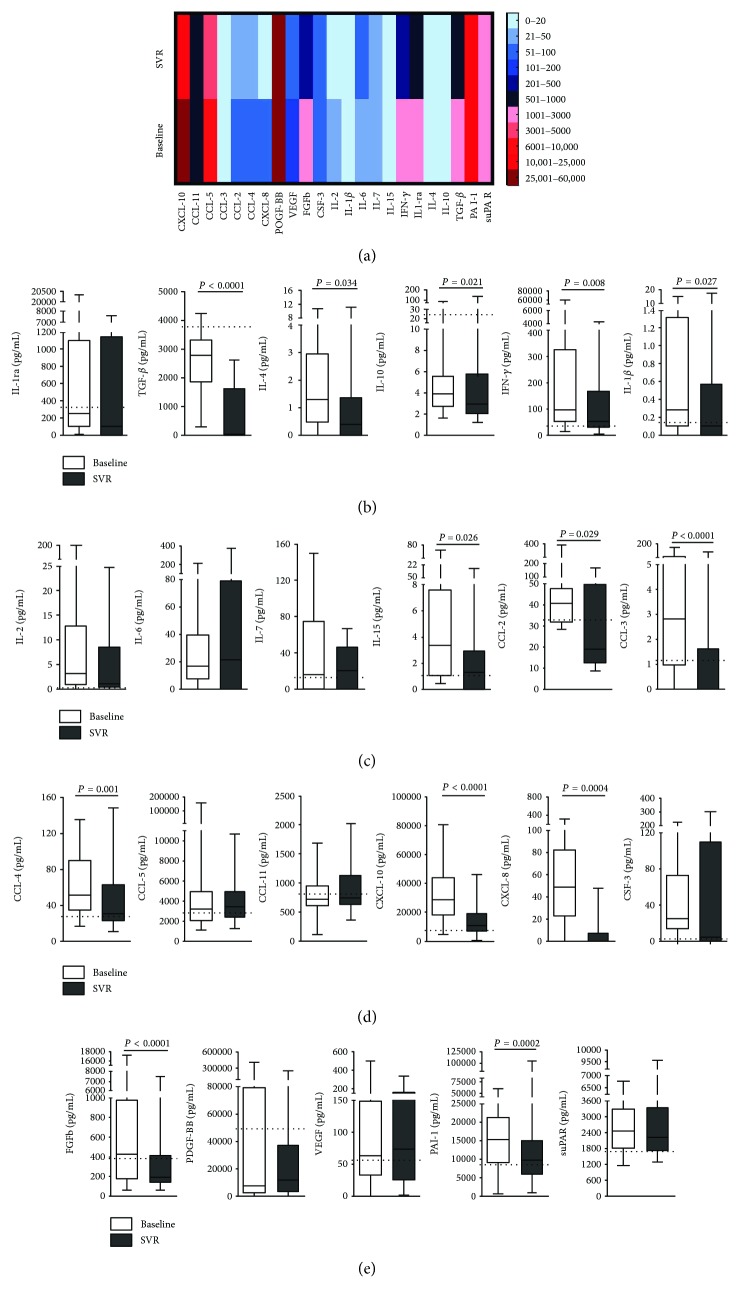
Modulation of cytokines after antiviral therapy represented through (a) heatmaps and box plot graphics of (b) IL-1ra, TGF-*β*, IL-4, IL-10, IFN-*γ*, IL-1*β*; (c) IL-2, IL-6, IL-7, IL-15, CCL-2, CCL-3; (d) CCL-4, CCL-5, CCL-11, CXCL-10, CXCL-8, CSF-3; and (e) FGFb, PDGF-BB, VEGF, PAI-1, and suPAR serum levels at baseline and SVR. Dashed lines mean values of HCV noninfected controls. Statistical significance was assessed by Wilcoxon test or paired *t*-test (*P* < 0.05).

A total of 23 immune mediators were analyzed, of which the mean serum levels of 18 presented a tendency to decrease after antiviral therapy, with the majority (11/18) reaching values comparable to the control group (Figure 1). Heatmap represents the expression pattern of immune mediators in chronic hepatitis C patients and their downregulation after DAA therapy (Figure 1(a)). This pattern was statistically significant in anti-inflammatory cytokines, IL-4 (*P* = 0.034), IL-10 (*P* = 0.021), and TGF-*β* (*P* < 0.0001) and proinflammatory cytokines, IFN-*γ* (*P* = 0.008), IL-1*β* (*P* = 0.027), and IL-15 (*P* = 0.026), as the same as for other molecules, FGFb (*P* < 0.0001) and PAI-1 (*P* = 0.002). Also, serum level decrease was significant for chemokines, CCL-2 (*P* = 0.029), CCL-3 (*P* < 0.0001), CCL-4 (*P* = 0.001), CXCL-8 (*P* = 0.0004), and CXCL-10 (*P* < 0.0001).

**Figure 2:**
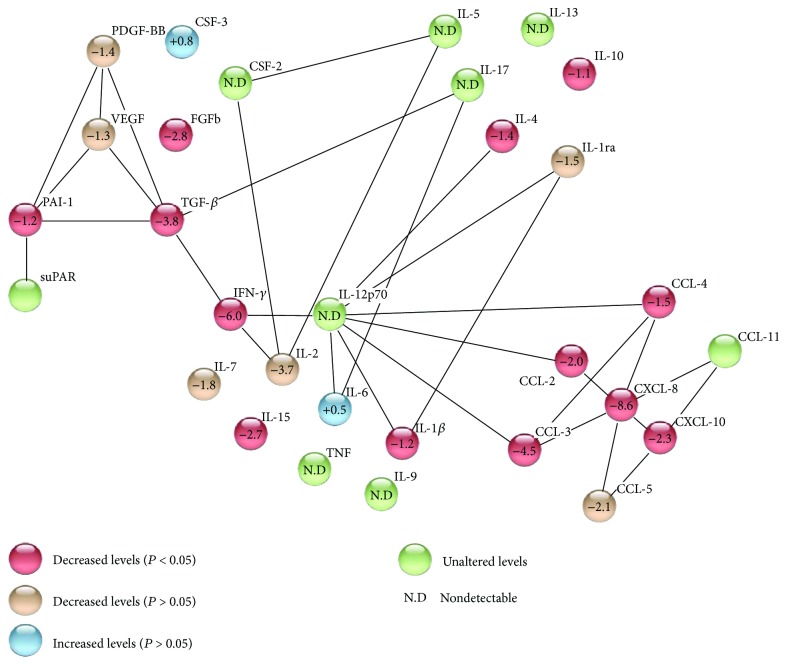
Network of protein-protein interaction, showing up- and downregulation presented by each studied cytokine after DAA therapy. This figure was built with software STRING v10.5.

Among the 18 biomarkers, the percentage of patients with decreasing levels after antiviral therapy was 82.5% for chemokines, 78.0% for anti-inflammatory cytokines, 72.4% for proinflammatory cytokines, and 77.9% for growth factors. Of all inflammatory biomarkers, only CSF-3 and IL-6 mean serum levels presented an increase (Figures 1(c), 1(d), and 2). However, this pattern only corresponds to a few number of patients (23.1% and 41.7%, resp.), and this was not statistically significant.

In our treatment center, only one patient that had undetectable viral load (<25 IU/mL) at the end of antiviral therapy presented detectable HCV-RNA (765 IU/mL) 12 weeks after the end of treatment, being characterized as a relapse patient. This individual was a 45-year-old female, infected by HCV genotype 3, with decompensated cirrhosis. Furthermore, this patient presented a clinical history of hypertension, dyslipidemia, hypothyroidism, and diabetes with notification of chronic venous leg ulcer. Unsurprisingly, hepatic parameters were increased after treatment when compared to baseline, such as ALT (34 to 48 U/L) and AST (50 to 93 U/L). Also, we observed an increase in APRI (0.83 to 2.39) and FIB-4 (2.24 to 5.56) scores and FibroScan values (22 to 51.4 kPa). At baseline, this relapse patient presented lower levels of immune mediators when compared to HCV-infected patients who achieved SVR. However, most biomarkers (CCL-3, CCL-4, CCL-5, CXCL-8, CXCL-10, IFN-*γ*, IL-1*β*, IL-6, IL-1ra, IL-10, PDGF-BB, and VEGF) were elevated after SOF-based antiviral therapy. The increase reached at least 2.31 times for IFN-*γ* (23.09 to 53.42 pg/mL) and at most 22.4 times for CXCL-8 (28.77 to 646.42 pg/mL).

The network of multiple protein-protein interactions involved in the pathogenesis of chronic hepatitis C is represented in Figure 2. Chemokines are related to each other and to proinflammatory cytokines, interacting with at least eight mediators. In general, we observed that proinflammatory cytokines and chemokines were mostly downregulated after IFN-free DAA antiviral therapy, and TGF-*β*, IFN-*γ*, CCL-3, and CXCL-8 presented the main decrease among all evaluated cytokines.

### 3.4. Chronic Hepatitis C Patient's Noninvasive Liver Parameters Improve after DAA Therapy

**Figure 3:**
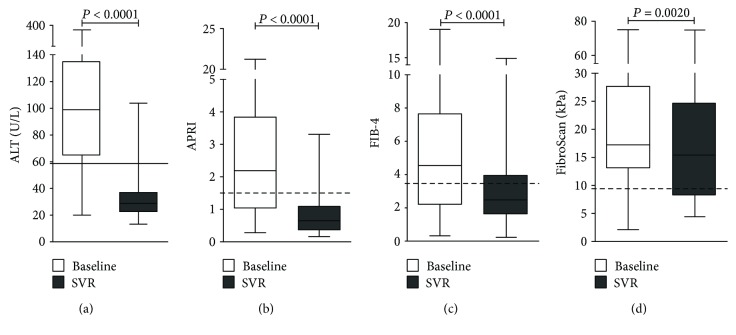
Evaluation of noninvasive hepatic parameters in chronic hepatitis C patients after DAA antiviral therapy. (a) ALT levels, (b) APRI and (c) FIB-4 indexes, and (d) FibroScan were decreased in SVR. Line: upper limit of normal values. Dashed lines: upper limit of F2 category. Statistical significance was assessed by Wilcoxon test or paired *t*-test (*P* < 0.05).

Regarding clinical laboratory tests, we observed an improvement in liver parameters after SOF-based therapy, especially in ALT (32.3 ± 15.3 U/L, *P* < 0.0001) and AST (31.1 ± 12.0 U/L, *P* < 0.0001) (Figure 3(a)). Importantly, albumin levels (3.8 ± 0.4 g/dL, *P* < 0.0001), PT (13.2 ± 3.6 seconds, *P* < 0.0001), and INR (1.2 ± 0.3, *P* < 0.0001) also ameliorate after treatment. On average, 97.2% of patients presented improvement in these noninvasive liver parameters after DAA therapy.

Furthermore, chronic hepatitis C patients also presented a significant decrease in APRI (*P* < 0.0001) and FIB-4 (*P* < 0.0001) scores after treatment (Figures 3(b) and 3(c)). Approximately 74% of patients presented a regression in liver injury classification at least in one applied methodology, while 34.5% of patients presented a regression in both APRI and FIB-4. Usually, patients' classification regressed from F3-F4 (advanced fibrosis-cirrhosis) to F2 (moderated fibrosis) after SOF-based treatment. Confirming these results, we also observed significant changes in FibroScan values (*P* = 0.002) after DAA therapy (Figure 3(d)), in which the majority (68.6%) of chronic hepatitis C patients presented reduction.

### 3.5. Inflammatory and Profibrogenic Biomarkers Are Differently Correlated with Liver Injury in Chronic Hepatitis C

We also analyzed the patterns of inflammatory and profibrogenic biomarker serum levels in patients with liver fibrosis (F0–F3), compensated cirrhosis (F4 child A), and decompensated cirrhosis (F4 child B or C), as well as the relationship between these immune mediators and liver injury, evaluated by ALT, APRI and FIB-4 indexes, and FibroScan, which are expressed in Tables 3 and 4, respectively.

Excluding CCL-11, higher levels of chemokines CCL-3, CCL-4, CCL-5, CXCL-8, and CXCL-10 were observed in compensated cirrhosis patients compared to fibrotic and decompensated cirrhosis patients (Table 3). Furthermore, serum levels of CCL-11 were significantly different between the three groups (*P* = 0.003), being more expressive in decompensated cirrhosis. Additionally, anti- and proinflammatory cytokine serum pattern in chronic hepatitis C patients did not present any statistically significant findings according to the liver disease stage (Table 3). Serum levels of growth factors revealed a decreasing pattern associated with an increase in liver disease severity; however, significant findings were only observed in FGFb (*P* = 0.019). Also, PAI-1 and suPAR serum levels were significantly different between the three stages of liver injury (*P* = 0.0007 and *P* = 0.0006, resp.).

The inverse correlation between serum levels of growth factors and stages of liver disease could be also observed when these biomarkers and noninvasive liver injury parameters were included in a linear regression analysis (Table 4). TGF-*β* serum levels were negatively correlated with PT (*P* = 0.027), APRI (*P* = 0.017), and FIB-4 (*P* = 0.001) indexes but not with FibroScan. FGFb was negatively correlated with INR (*P* = 0.04) and positively associated with albumin levels (*P* = 0.026). Moreover, serum levels of IL-2 (*P* = 0.039), IL-15 (*P* = 0.004), and IL-1ra (*P* = 0.008) were positively correlated with FIB-4 index, ALT, and AST levels, respectively. We identified negative correlations between chemokines: CXCL-10 with PT (*P* = 0.015) and INR (*P* = 0.004), besides CCL-11 with albumin levels (*P* = 0.005).

In addition to the significant difference (*P* = 0.0006) observed in serum levels of suPAR between fibrosis and cirrhosis groups of patients (Table 3), positive correlations with most of noninvasive measures of liver injury were observed: albumin levels (*P* = 0.0001), APRI (*P* = 0.037), FIB-4 (*P* = 0.010), and FibroScan (*P* = 0.001) (Table 4).

## 4. Discussion

In most cases of HCV infection, the immune system is unable to eradicate the virus, leading to the establishment of a chronic disease, characterized by persistent inflammation and liver fibrosis. In this study, we investigated the cytokine profile after SOF-based therapy in chronic hepatitis C patients aiming to verify if DAA-induced virus eradication was followed by restoration in cytokine levels and improvement in liver function. Therefore, 88 chronic hepatitis C patients treated with IFN-free SOF-based regimens were followed, and serum levels of 30 circulating biomarkers were assessed at baseline and 12 weeks after the end of treatment. HCV noninfected individuals were used as controls. This is a relevant study that demonstrates cytokine and chemokine modulation, besides liver function restoration in HCV chronic-infected patients after antiviral therapy.

Most inflammatory mediators play an important role in the development of liver inflammation as well as disease progression in infected HCV patients. When compared to the control group, the patients presented an upregulation pattern restricted to serum levels of CCL-4, CXCL-10, IFN-*γ*, PAI-1, and suPAR. Recently, Hengst et al. [11] have shown upregulation in levels of CXCL-10, IL-12p40, IFN-*α*2, LTA, TRAIL, and IL-18 and downregulation of IL-17, IL-1*β*, FGFb, PDGF-BB, IFN-*γ*, and IL-4 in patients persistently infected with HCV as compared to healthy individuals. In another study, chronic hepatitis C patients presented elevated serum levels of IL-10, IFN-*γ*, IL-6, and IL-4, while IL-1, IL-2, IL17, IL-22, IL-13 TNF, IL-12p70, and IL-15 levels were lower in patients compared to healthy controls [12]. In contrast, the levels of IFN-*γ* were described to be lower in another population of chronic hepatitis C patients compared to controls, while IL-4 and IL-10 were higher [29]. These data exemplify the disturbance in the immune system that had already been described in HCV persistent infection. The controversial findings can be attributed to the ability to trigger an efficient immune response against HCV infection in each patient. Besides, age, gender, viral load, and liver disease status are also considered interference factors in HCV infection pathogenesis.

After antiviral therapy, our results demonstrated significant changes in serum levels of CCL-2, CCL-3, CCL-4, CXCL-8, CXCL-10, IL-4, IL-10, TGF-*β*, IFN-*γ*, IL-1*β*, IL-15, FGFb, and PAI-1, in association with viral clearance. The levels of some biomarkers posttreatment were decreased in our study population, reaching levels similar to controls, mainly chemokines CCL-2, CCL-3, and CXCL-10; proinflammatory cytokine IL-1*β*; and growth factor FGFb. Until this moment, evidences have suggested that the disrupted pattern of soluble inflammatory mediators induced by HCV infection remains even after viral clearance with DAA treatment, indicating that the inflammatory changes are not fully reversible upon viral clearance [11]. Increased levels of TGF-*β* and IL-15 were found in patients who spontaneously cleared HCV infection, while those patients with persistent infection presented elevated levels of CCL-5 and IL-8 [30]. Considering chemokine set, a decrease in CCL-2, CCL-4, and CXCL-10 levels were reported in HCV genotype 2- and 3-infected patients after antiviral treatment, with lower levels of CCL-4 at baseline associated to an early response to treatment [24]. A putative mechanism in which DAA-induced viral clearance restores immune response has been demonstrated by an increase in T cell count, downregulation of negative costimulatory molecules, and restoration of cytolytic activity [31].

Elevated frequency of regulatory T cells (Treg) in the peripheral blood from chronic hepatitis C patients has been described [32]. Long-term maintenance of Treg cells is involved in the chronic progression of disease, whereas spontaneous recovery in the acute stage was associated with loss of the suppressive function of Treg cells [33]. This data suggests that Treg cells, which secreted IL-10 and TGF-*β* anti-inflammatory cytokines, are determinant elements in the spontaneous resolution or progression of HCV infection to chronicity [33]. HCV-infected patients when submitted to IFN treatment revealed elevated frequency of Treg and CD8+ T cells, while they appeared to present decrease of TGF-*β* serum levels [34]. In this study, we observed a decrease of IL-10 and TGF-*β* serum levels after IFN-free DAA therapy that could be explained by Treg cell modulation after treatment, though studies with focus in Treg or distinct Treg subpopulations need to be performed in HCV-infected patients after treatment to clear the contribution of these cells in viral control.

It is well known that cytokines can participate in repair/regeneration process, required after resolution of infection. In fact, IL-6, also known as hepatocyte-stimulating factor, is a pleiotropic cytokine secreted during inflammatory conditions in response to liver injury [17]. In this study, IL-6 levels were not significantly altered after antiviral therapy. Confirming our results, IL-6 serum levels were not altered after DAA treatment in another Brazilian cohort with chronic hepatitis C patients [20]. Interestingly, another Brazilian study suggests that IL-6 and TNF polymorphisms might lead to a distinct range of proinflammatory cytokines [23]. In this study, a multiple-locus analysis was not performed, and thus we do not exclude the possibility that IL-6 serum levels are maintained after treatment due to its involvement in liver repair process after HCV elimination.

Our cohort of patients is characterized by elevated prevalence of female, patients with cirrhosis, and previously treated. The Brazilian guideline for HCV treatment recommended priority in therapy for patients with advanced stages of liver disease [2]. The overall downregulation of fibrotic and inflammatory biomarkers is accompanied by an improvement in liver function, which could be noticed by an expressive decrease in noninvasive APRI and FIB-4 scores, liver stiffness values, and laboratory parameters, such as ALT, albumin, PTT, and INR. These data suggest that with HCV eradication, inflammation, and fibrosis could regress. Previous studies have demonstrated a decrease, after HCV eradication, in noninvasive liver fibrosis measurements, such as liver stiffness [14, 15, 35–37] and noninvasive indexes APRI [35, 36] and FIB-4 [35]. Likewise, improvements in inflammation were also observed in histological findings performed by liver biopsy [13].

Along, we demonstrated correlations between cytokines and liver disease severity in HCV-infected patients. TGF-*β* and suPAR were moderately correlated (negative and positive correlation, resp.) with fibrosis measurement parameters. Soluble uPAR is a well-described and stable prognostic biomarker in inflammatory and infectious diseases [38], in which the correlation with liver disease severity was previously described [39, 40]. On the other hand, serum TGF-*β* may not be a major immune mediator in later stages of viral persistent infection [41], although its expression has been demonstrated, mostly in the liver tissue of HCV chronic-infected patients [42]. In a HCV humanized mice model, the normalization of HCV-induced disturbance in both the peripheral blood and liver after DAA therapy was confirmed [43]. Despite that, a long-term follow-up is necessary to clarify main potential changes in these processes, mainly in the liver immunopathology, in order to confirm a possible regression/stability of inflammation and/or fibrosis.

In our treatment center, patients upon DAA treatment have excellent SVR rate [4]. Only one patient showed viral relapse after the end of treatment. This patient was infected with HCV genotype 3 and had decompensated cirrhosis. In fact, it is known that HCV genotype 3-infected groups are hard to cure, even with the newer DAAs [44]. Probably, this patient relapsed due to the therapeutic regimen of 12 weeks recommended by Brazilian guidelines [2] instead of 24 weeks to genotype 3. A limitation of this study was the nonstratification of patients according to treatment outcome (SVR versus relapse/nonresponder patients) exactly due to the high rate of SVR, although we could notice in our treatment center that antiviral therapy based on DAA improved patient's quality of life, highlighted by the high rates of SVR associated with a safe profile and good tolerability, even in patients with advanced liver disease [4].

In summary, our data suggest that IFN-free SOF-based treatment induced viral control leading to the downregulation of circulating proinflammatory cytokines, chemokines, and growth factors, what was observed in association with improvement in liver function.

## 5. Conclusion

In conclusion, our data provide evidences that viral clearance induced by DAA antiviral therapy leads to the downregulation and restoration of circulating cytokines in addition to improvement in liver function. These results indicate a possibility of a long-term reversal in inflammation and fibrosis processes.

## Figures and Tables

**Table 1:** Baseline demographic and clinical characteristics of chronic hepatitis C patients.

Characteristics	*n* = 88
Age in years, mean ± SD (min–max)	59.4 ± 8.95 (23.0–73.0)
Gender, *n* (%)	
Female/male	61 (69.32)/27 (30.68)
HCV genotype, *n* (%)	
1	71 (80.68)
3	16 (18.18)
4	1 (1.14)
HCV viral load (IU/mL), mean (±SD)	17.7 × 10^5^ (±34.7 × 10^5^)
Liver disease status, *n* (%)	
F0–F3	17.0 (19.32)
F4	
Child A	58.0 (65.91)
Child B or C	13.0 (14.77)
ALT (U/L), median (IQR)	99.0 (64.5–134.8)
AST (U/L), median (IQR)	73.5 (46.2–117.0)
Albumin (g/dL), median (IQR)	3.45 (3.1–3.8)
PT (seconds), median (IQR)	13.0 (12.2–14.3)
INR, median (IQR)	1.18 (1.11–1.3)
Previous therapy, *n* (%)	50.0 (56.82)
Current therapy, *n* (%)	
SOF + DCV	12.0 (13.64)
SOF + DCV + RBV	55.0 (62.50)
SOF + SMV	8.0 (9.09)
SOF + SMV + RBV	13.0 (14.77)
Therapy duration, *n* (%)	
12 weeks	75.0 (85.23)
24 weeks	13.0 (14.77)
Therapy outcome, *n* (%)	
SVR	87.0 (98.86)
Relapse	1.0 (1.14)

Data are expressed as mean (±SD) or *n* (%). ALT: alanine aminotransferase; AST: aspartate aminotransferase; DCV: daclatasvir; GGT: gamma-glutamyl transferase; HCV: hepatitis C virus; IQR: interquartile range; RBV: ribavirin; SD: standard deviation; SOF: sofosbuvir; SMV: simeprevir.

**Table 2:** Cytokine range in serum chronic hepatitis C patients compared to HCV noninfected controls.

	Chronic hepatitis C	Control group	*P* value
Chemokines			
CCL-3	2.8 (0.9–16.6)	1.3 (0.6–1.6)	NS
CCL-4	50.9 (34.5–89.2)	24.9 (18.5–28.8)	**0.001**
CCL-5	3186 (2032–4881)	2625 (2025–3370)	NS
CCL-11	722.7 (597.7–944.5)	788.9 (261.1–1235.0)	NS
CXCL-10	29,385 (18,159–44,260)	9120 (4075–10,196)	**0.0001**
Pro-inflammatory			
IFN-*γ*	98.4 (53.4–324.8)	33.3 (14.5–51.8)	**0.002**
IL-1*β*	0.3 (0.1–0.5)	0.2 (0.03–0.2)	NS
Anti-inflammatory			
IL-10	4.0 (2.7–5.6)	4.6 (2.4–41.2)	NS
IL-1ra	246.3 (98.0–1102.0)	307.5 (46.0–603.1)	NS
TGF-*β*	2781 (1856–3302)	3821 (3239–4098)	**0.004**
Growth factors			
FGFb	423.9 (167.7–976.3)	314.9 (115.4–435.9)	NS
PDGF-B	7932 (2183–78,666)	51,254 (28,252–71,775)	NS
VEGF	65.4 (33.3–149.3)	73.5 (31.4–4269.0)	NS
Others			
PAI-1	15,325 (8890–21,330)	5038 (3276–13,210)	**0.008**
suPAR	2478 (1772–3320)	1575 (1283–1878)	**0.023**

Data are presented as median (interquartile range). NS: nonsignificant. HCV-noninfected individuals who presented detectable levels of CCL-2, CXCL-8, and IL-15 were not enough for analysis. Statistical significance was assessed by Mann–Whitney or unpaired *t*-test (*P* < 0.05). The bold numbers indicate *P* values < 0.05.

**Table 3:** Expression pattern of cytokines in chronic hepatitis C patients with different liver disease status.

	F0–F3	F4 child A	F4 child B or C	*P* value
Chemokines				
CCL-3	3.1 (1.8–8.5)	12.1 (0.8–28.8)	2.1 (1.3–11.8)	NS
CCL-4	46.1 (31.8–56.9)	74.9 (38.0–111.7)	49.4 (33.4–79.7)	NS
CCL-5	2526 (1962–3828)	3978 (1970–5719)	3089 (1874–4779)	NS
CCL-11	722.7 (620.7–869.1)	466.5 (347.4–845.2)	1004 (717.5–1625)	**0.003**
CXCL-8	36.6 (24.8–60.3)	66.7 (40.5–90.6)	62.7 (26.7–234.7)	NS
CXCL-10	29,024 (17571–38,389)	29,385 (14,847–33,201)	27,127 (14,624–52,988)	NS
Proinflammatory				
IFN-*γ*	98.4 (48.6–194.3)	74.9 (53.4–1417.0)	92.5 (46.8–450.6)	NS
IL-1*β*	0.1 (0.1–0.8)	0.7 (0.3-4.1)	0.3 (0.1-2.7)	NS
IL-15	2.2 (1.0–10.0)	2.9 (0.5–7.6)	4.8 (1.3–27.5)	NS
Anti-inflammatory				
IL-10	5.3 (3.3–6.0)	2.9 (2.1–3.8)	4.5 (2.7–11.6)	NS
IL-1ra	215.5 (73.8–468.2)	293.3 (151.9–1894.0)	207.9 (55.4–1660.0)	NS
TGF-*β*	3146 (2707–3935)	2300 (1856–2879)	2468 (1629–3286)	NS
Growth factors				
FGFb	478.2 (125.5–1595.0)	447.4 (302.4–885.1)	143.1 (139.9–187.7)	**0.019**
PDGF-BB	29,579 (2670–133,040)	7932 (2250–23,508)	3198 (133.1–62,475)	NS
VEGF	62.9 (32.8–119.3)	80.3 (41.0–271.8)	58.0 (43.2–191.5)	NS
Others				
PAI-1	20,154 (15,668–23,239)	9927 (5816–13,729)	19,469 (15,882–25,636)	**0.0007**
suPAR	1759 (1587–2108)	2441 (2006–2992)	3365 (2888–4062)	**0.0006**

Data are presented as median (interquartile range). NS: nonsignificant. The analysis of CCL-2 was not performed due to insufficient number of patients. Statistical significance was assessed by ANOVA with Bonferroni's posttest or Kruskal-Wallis test with Dunn's posttest (*P* < 0.05). The bold numbers indicate *P* values < 0.05.

**Table 4:** Correlation between cytokines and noninvasive methodologies for liver injury measure.

		ALT	Albumin	APRI	FIB-4	FibroScan
CCL-2	*r*	−0.028	−0.057	0.060	0.351	0.466
	*P*	NS	NS	NS	NS	NS
CCL-3	*r*	−0.250	0.241	−0.264	−0.194	−0.310
	*P*	NS	NS	NS	NS	NS
CCL-4	*r*	0.143	−0.029	−0.080	0.071	0.063
	*P*	NS	NS	NS	NS	NS
CCL-5	*r*	0.043	0.041	0.119	0.146	0.199
	*P*	NS	NS	NS	NS	NS
CCL-11	*r*	0.040	−**0.454**	0.032	0.066	0.135
	*P*	NS	**0.005**	NS	NS	NS
CXCL-8	*r*	−0.020	−0.182	0.229	0.420	0.018
	*P*	NS	NS	NS	NS	NS
CXCL-10	*r*	0.029	−0.050	−0.052	−0.012	−0.235
	*P*	NS	NS	NS	NS	NS
IFN-*γ*	*r*	0.294	−0.058	0.070	0.041	−0.019
	*P*	NS	NS	NS	NS	NS
IL-1*β*	*r*	0.148	−0.201	0.225	0.258	0.249
	*P*	NS	NS	NS	NS	NS
IL-2	*r*	0.457	−0.521	0.451	**0.577**	−0.027
	*P*	NS	NS	NS	**0.039**	NS
IL-6	*r*	−0.228	−0.311	0.048	0.381	−0.190
	*P*	NS	NS	NS	NS	NS
IL-7	*r*	0.500	0.355	0.429	0.250	0.500
	*P*	NS	NS	NS	NS	NS
IL-15	*r*	**0.535**	−0.298	0.495	0.438	−0.066
	*P*	**0.04**	NS	NS	NS	NS
IL-4	*r*	0.195	−0.085	0.042	−0.079	−0.095
	*P*	NS	NS	NS	NS	NS
IL-10	*r*	0.250	0.000	−0.100	−0.183	−0.204
	*P*	NS	NS	NS	NS	NS
IL-1ra	*r*	0.178	−0.111	0.196	0.204	0.289
	*P*	NS	NS	NS	NS	NS
TGF-*β*	*r*	−0.043	0.281	−**0.395**	−**0.521**	−0.351
	*P*	NS	NS	**0.017**	**0.001**	NS
CSF-3	*r*	0.494	−0.162	0.213	0.231	0.144
	*P*	NS	NS	NS	NS	NS
FGFb	*r*	0.040	**0.295**	−0.154	−0.138	−0.195
	*P*	NS	**0.026**	NS	NS	NS
PDGF-BB	*r*	0.024	0.328	−0.319	−**0.365**	−0.330
	*P*	NS	NS	NS	**0.028**	NS
VEGF	*r*	0.118	0.036	0.168	0.189	0.154
	*P*	NS	NS	NS	NS	NS
PAI-1	*r*	0.161	−0.238	0.026	−0.061	−0.069
	*P*	NS	NS	NS	NS	NS
suPAR	*r*	0.089	−**0.531**	**0.350**	**0.424**	**0.542**
	*P*	NS	**0.001**	**0.037**	**0.010**	**0.001**

*r*: Spearman's or Pearson's coefficients for non-parametric and parametric parameters, respectively. NS: nonsignificant. *P*: indicate significant value < 0.05. The bold numbers indicate *P* values < 0.05.

## Abbreviations

ALT: Alanine aminotransferase
APRI: Platelet ratio index
AST: Aspartate aminotransferase
CCL: Chemokine C-C ligand
CSF: Colony stimulation factor
CXCL: Chemokine X-C ligand
DAA: Direct-acting antiviral agents
DCV: Daclatasvir
FGFb: Basic fibroblast growth factor
FIB-4: Fibrosis-4 index
GGT: Gamma-glutamyl transferase
HCV: Hepatitis C virus
IFN: Interferon
IL: Interleukin
INR: International normalized ratio
PAI-1: Plasminogen activator inhibitor type 1
PDGF-BB: Platelet-derived growth factor BB
PT: Prothrombin time
RBV: Ribavirin
SMV: Simeprevir
SOF: Sofosbuvir
suPAR: Soluble urokinase-type plasminogen activator receptor
SVR: Sustained virological response
TGF-*β*: Transforming growth factor beta
TNF: Tumor necrosis factor
VEGF: Vascular endothelial growth factor.
